# VFedA: A Vertical Federated Learning Framework with Relational Alignment for Cross-Regional False Data Injection Attack Detection in Smart Grids

**DOI:** 10.3390/s26196336

**Published:** 2026-10-08

**Authors:** Binyuan Yan, Guozhi Yang, Xin Che, Xiaohan Huang

**Affiliations:** 1 Guizhou Power Grid Digital Intelligence Center, Guizhou Power Grid Co., Ltd., Guiyang 550081, China; yanby@gz.csg.cn; 2 College of Computer Science and Technology, Guizhou University, Guiyang 550025, China; gs.xhhuang23@gzu.edu.cn; 3 College of Control Science and Engineering, Zhejiang University, Hangzhou 310027, China

**Keywords:** false data injection attack, smart grid security, vertical federated learning, cross-regional feature fusion, relational alignment, spatiotemporal representation learning

## Abstract

Large-scale smart grids have introduced significant challenges for false data injection attack (FDIA) detection due to the distributed nature of power system operation and the limited observability of individual regions. Existing horizontal federated learning approaches enable collaborative model training without sharing raw data; however, they rely on local information and often fail to capture the global operational characteristics needed to detect sophisticated attacks. To address this limitation, the present paper proposes VFedA, a federated vertical relational alignment framework for FDIA detection via cross-regional feature fusion. Specifically, each regional client employs a self-supervised spatiotemporal contrastive learning encoder to extract local representations from measurements. The extracted representations are uploaded to a control center and concatenated to construct global feature representations for attack detection. In order to mitigate inconsistencies across heterogeneous regional feature spaces, a relational alignment mechanism is introduced to enforce structural consistency by aligning similarity matrices derived from local representations. The proposed approach enables effective cross-regional information fusion while preserving data privacy. Extensive experiments are conducted on IEEE 30-bus and IEEE 118-bus benchmark systems. The results demonstrate that VFedA consistently outperforms conventional horizontal federated learning methods and achieves performance comparable to centralized detection models.

## 1. Introduction

Cyber–physical smart grids have fundamentally transformed the way electricity is generated, transmitted, and distributed. Such grids integrate advanced communication technologies, automated control systems, and pervasive sensing infrastructures. Supervisory control and data acquisition (SCADA) systems are equipped with remote terminal units and phasor measurement units. As a result, smart grids can achieve real-time monitoring, adaptive control, and data-driven decision-making [1]. However, this deep coupling between information technology and physical power systems also introduces cybersecurity vulnerabilities [2,3]. Among the various cyber threats targeting smart grids, false data injection attacks (FDIA) pose one of the most serious challenges [4].

FDIA refers to a class of cyberattacks in which adversaries manipulate measurement data while complying with the physical constraints of the power grid. This manipulation allows them to evade traditional bad data detection (BDD) mechanisms [5]. Attackers inject carefully crafted attack vectors that satisfy the consistency relationships between measurement data and state estimation models. As a result, FDIAs can shift state estimation results while leaving detection residuals unchanged. This gives such attacks exceptional stealth and destructive potential. The consequences of a successful FDIA can be severe, ranging from compromised data integrity and unreliable system operation to cascading failures and significant economic losses. The 2015 Ukraine power grid attack is a real-world example [6].

In response to the growing threat of FDIAs, considerable research has focused on developing detection methods. Traditional model-based approaches such as residual-based BDD techniques are inherently limited, primarily addressing random measurement errors rather than targeted malicious alteration. Data-driven methods leveraging machine learning have emerged as promising alternatives. These include principal component analysis, deep neural networks, ensemble classifiers, and graph neural networks, which have demonstrated varying degrees of effectiveness [7,8,9]. Despite these advances, existing detection approaches face a fundamental limitation in that they typically assume centralized data availability; in such approaches, measurement data from all buses must be uniformly collected and used for model training. This assumption is increasingly untenable in modern multi-regional smart grids in which data are distributed across multiple operators and regions. In addition, privacy regulations or commercial sensitivities often preclude the sharing of raw operational data [10].

Federated learning is a promising paradigm for addressing the challenge of collaborative training in smart grid cybersecurity while preserving privacy [11]. Federated learning enables distributed training of machine learning models without the need for participants to share their raw data; thus, multiple grid operators can collaboratively build robust FDIA detectors while preserving data privacy [12]. Most existing federated learning approaches for FDIA detection adopt a horizontal federated learning (HFL) framework. In HFL, data from different clients occupy the same feature space but different sample spaces. HFL enables collaborative model training across regions; however, each client’s local model relies on its own measurement data, and so lacks access to the global operational characteristics needed in order to detect sophisticated cross-regional attacks. As a result, HFL-based detectors often suffer from limited observability and cannot fully capture the complex spatiotemporal dependencies that span multiple regions.

This paper proposes VFedA to address these limitations. VFedA is a vertical federated relational alignment framework for FDIA detection. It enables cross-regional feature fusion while preserving data privacy. Unlike HFL, vertical federated learning is applicable when different parties have distinct feature spaces for the same set of samples. This is a natural fit for multi-regional smart grids, in which each region observes the same time instances but from different geographical and topological perspectives. The proposed VFedA framework comprises four key components. First, each regional client employs a self-supervised spatiotemporal contrastive learning encoder that extracts local representations from power system measurements without requiring labeled data. Second, these local representations are uploaded to a control center, where they are concatenated to form global feature representations that capture cross-regional operational states. Third, a lightweight detection model is trained at the control center. It uses only a small fraction of labeled samples. Fourth and most critically, a relational alignment mechanism is introduced to enforce structural consistency across heterogeneous regional feature spaces. This is achieved by aligning similarity matrices derived from local representations. As a result, the alignment mechanism mitigates inconsistencies in feature space arising from independently trained local encoders. The main contributions of this paper are threefold:

- We propose a vertical federated learning framework for FDIA detection. It enables effective cross-regional information fusion while preserving data privacy, and also addresses the limited observability challenge inherent in conventional HFL approaches.
- We introduce a relational alignment mechanism to enforce structural consistency across heterogeneous regional feature spaces. This is achieved by aligning similarity matrices derived from local representations. The mechanism enables more stable and discriminative joint representations for global detection.
- We conduct extensive experiments on the IEEE 30-bus and IEEE 118-bus benchmark systems. The results demonstrate that VFedA consistently outperforms conventional HFL methods. VFedA also achieves performance comparable to that of centralized detection models.

The remainder of this paper is organized as follows: Section 2 provides the related work; system preliminaries for the later derivations are given in Section 3; Section 4 formulates the problem solved by this work; Section 5 details the design of the VFedA method, including the overall framework and the convergence analysis; Section 6 presents experimental evaluation results; finally, Section 8 concludes the paper.

## 2. Related Work

This section reviews the existing literature that is most relevant to our work. It is organized into three main streams: (1) false data injection attack detection methods in smart grids, (2) federated learning for power system security, and (3) contrastive learning and self-supervised representation learning for anomaly detection.

### 2.1. False Data Injection Attack Detection

False data injection attacks were first formally introduced by Liu et al. [13]. They have since become one of the most critical cybersecurity threats to modern smart grids. Unlike conventional cyberattacks that cause immediate operational disruptions, FDIAs exploit the physical constraints of power systems. They construct malicious measurement vectors that evade traditional bad data detection (BDD) mechanisms, and can also induce substantial errors in state estimation. Such attacks are stealthy. They can cause cascading failures and widespread blackouts, which has motivated extensive research on effective detection methodologies [14].

Existing FDIA detection approaches can be broadly categorized into model-based and data-driven methods. Model-based techniques rely on precise knowledge of system topology and parameters. They employ residual-based statistical tests or Kalman filtering to identify anomalies [15]. However, these methods are sensitive to parameter uncertainties and model inaccuracies, limiting their practical applicability in dynamically evolving large-scale power systems.

Data-driven methods have gained substantial traction in recent years. They leverage machine learning techniques to learn discriminative patterns from measurement data and do not require explicit system models. A comprehensive survey by Irfan et al. [16] systematically reviewed more than 40 major research contributions on FDIA detection and localization, highlighting the predominant role of data-driven techniques and mathematical modeling in the proposed approaches. Another comprehensive survey in 2025 further established a taxonomy of machine learning-based FDIA detection methods. It emphasized the utility of graph neural networks, autoencoders, and federated learning in addressing sub-problems such as privacy preservation, generalized detection, and attack localization [17].

Within the data-driven paradigm, deep learning architectures have demonstrated particular promise. Spatiotemporal transformer networks have been proposed for FDIA detection. These capture both spatial correlations across buses and temporal dependencies in measurement sequences. Graph neural networks are especially well suited for power system applications due to the inherent graph structure of the grid topology [18]. For instance, graph autoencoders exploit spatial correlations between connected buses, allowing them to efficiently detect and mitigate FDIA effects [18].

Despite these advances, most existing data-driven FDIA detection methods operate under a centralized paradigm. They assume that measurement data from all buses can be aggregated at a central location for model training, which becomes increasingly problematic in multi-regional smart grids. In such grids, data privacy concerns, communication constraints, and regulatory barriers prevent the sharing of raw operational data across regional boundaries. These limitations have motivated the exploration of privacy-preserving collaborative learning frameworks, among which federated learning has emerged as the most promising paradigm.

### 2.2. Graph Neural Network-Based Detection

Graph neural networks are especially well suited for power system applications due to the inherent graph structure of the grid topology. GNNs can encode the physical structure of the power grid, capturing spatial correlations between connected buses. A 2026 survey found that GNNs showed superior performance in detecting false data injection attacks by encoding the physical structure of the power grid, while transformer-based models excelled in detecting electricity theft attacks by capturing long-range temporal dependencies [19].

Several recent works have advanced GNN-based FDIA detection. A spatiotemporal graph neural network (ST-GNN) with adaptive mechanisms was proposed for FDIA detection and localization, incorporating multi-head attention, dynamic graph learning, and adaptive weight adjustments. Experiments on the IEEE 14-bus test system demonstrated that the adaptive ST-GNN significantly outperforms the basic ST-GNN [20]. A graph convolutional network (GCN) combined with LSTM has been applied to detect data integrity cyberattacks on STATCOM, exploiting both spatial and temporal correlations in power system data [21].

Distributed FDIA detection and localization based on heterogeneous data feature extraction and reconstruction of graph convolutional networks have been explored for microgrids [22]. A recursive variational graph autoencoder (RVGAE) was proposed for locational FDIA detection in smart grids, exploiting spatial correlations between connected buses to efficiently detect and mitigate FDIA effects [18]. A graph contrastive learning method for power system state estimation was proposed by Li et al. [23]. FedCLD extends contrastive learning to a federated setting, allowing for the detection of stealthy attacks with unlabeled data [4].

### 2.3. Federated Learning for Power System Security

Federated learning was first introduced by McMahan et al. [24]. It enables distributed training of machine learning models across multiple clients, capturing spatial correlations among data while preserving privacy and leveraging collective intelligence. The application of federated learning to smart grid security has attracted considerable research attention. Recent studies have comprehensively surveyed how FL can support secure and decentralized anomaly detection. Studies have also analyzed how FL can mitigate attacks such as FDIA and DDoS on smart grid infrastructure [25].

The majority of existing FL-based FDIA detection approaches adopt the HFL paradigm. In this paradigm, different regions or substations share the same feature space but have distinct sample sets. One distributed detection and mitigation framework based on federated learning incorporated a graph autoencoder to capture spatial correlations among power load profiles [8]. This approach was validated on the IEEE 57-, 118-, and 300-bus systems, demonstrating the effectiveness of FL-based distributed FDIA detection [8]. More recently, a protected federated deep learning scheme (ProFed) combined the Federated Averaging (FedAvg) algorithm with pretrained enhanced spatiotemporal graph neural networks and Cheon–Kim–Kim–Song (CKKS) homomorphic encryption. This approach helps to protect sensitive information during training [26].

Other federated learning-based schemes use hybrid deep neural network architectures incorporating LSTM and GCN layers to exploit both temporal and spatial patterns for FDIA detection. To address the challenge of non-IID data distributions across clients, clustered federated learning approaches such as FedClusAvg have been developed. These approaches improve detection accuracy under heterogeneous data distributions while reducing communication overhead [4].

While HFL has been extensively explored, vertical federated learning has also been studied. In VFL, different participants share the same sample space but have different feature spaces. This is particularly relevant for multi-regional power systems. In these systems, different regions naturally collect heterogeneous measurements from the same temporal samples; examples include voltage magnitudes, phase angles, and power injections. Kesici et al. [10] proposed an original vertical federated learning framework based on split learning for FDIA detection in distribution networks [10]. In their approach, grid edge-side entities extract spatial features through an attention-based deep learning model. The server then extracts temporal features using a Bi-LSTM model.

However, existing vertical federated learning approaches for FDIA detection face several limitations. First, they typically assume the availability of labeled data for supervised training. In real-world power systems, however, attack labels are scarce and costly to obtain. Second, they do not adequately address the challenge of feature space heterogeneity across independently trained regional encoders. This is a fundamental issue when regions employ different model architectures or training procedures. Third, existing methods lack mechanisms to enforce structural consistency across regional representations. Such consistency is essential for effective feature fusion. Our proposed VFedA framework directly addresses these gaps by introducing self-supervised contrastive learning for label-free representation extraction together with a relational alignment mechanism that enforces structural consistency across heterogeneous regional feature spaces.

### 2.4. Contrastive Learning and Self-Supervised Representation Learning

The scarcity of labeled data in power system security applications has motivated the adoption of self-supervised and contrastive learning approaches. These paradigms enable models to learn meaningful representations from unlabeled data. They do so by solving pretext tasks that capture inherent data structures.

Contrastive learning has emerged as a powerful technique for learning discriminative representations. It maximizes agreement between differently augmented views of the same sample while minimizing agreement between views of different samples [23]. In power system applications, contrastive learning has been applied to electricity load anomaly detection. Multi-feature fusion and contrastive learning frameworks integrate temporal and spatial features in order to jointly detect anomalies [27].

Specifically for FDIA detection, semi-supervised approaches leveraging temporal-frequency contrastive learning have been proposed for attack localization. Federated contrastive learning frameworks (FedCLD) have also been developed for detecting stealthy attacks on smart grids using unlabeled data. These approaches demonstrate the potential of contrastive learning to address the label scarcity challenge in power system security [4]. In representation learning, power system measurements exhibit rich spatiotemporal patterns. Spatial correlations arise from the grid topology and power flow constraints, while temporal dependencies reflect the dynamic evolution of system states. Recent research has focused on developing spatiotemporal representation learning methods that capture both dimensions simultaneously [28]. Spatiotemporal contrastive graph convolutional networks integrate graph convolutional network encoders to capture spatial dependencies, while temporal convolutional networks are used to model electromechanical dynamics. This approach has demonstrated robustness to topology variations.

Despite these advances, existing contrastive and self-supervised learning methods for power systems have primarily been developed for centralized settings. In these settings, data from all sources can be pooled for representation learning. The integration of self-supervised representation learning with federated learning remains largely unexplored. Our VFedA framework fills this gap, enabling each regional client to independently learn spatiotemporal representations through self-supervised contrastive learning; meanwhile, the relational alignment mechanism ensures cross-regional consistency without requiring label information.

### 2.5. Summarization and Comparison

The above studies demonstrate that substantial progress has been made in FDIA detection, federated learning for smart grid security, and self-supervised representation learning. However, these research directions have largely evolved independently. Centralized FDIA detection methods can exploit comprehensive system information, but require access to raw measurements. Conventional HFL methods preserve data locality, but generally restrict each client to its local feature space. Existing VFL-based FDIA detection approaches provide a means of cross-party feature collaboration; however, they remain limited by their reliance on labeled training data and also provide insufficient treatment of heterogeneous feature spaces. Meanwhile, existing self-supervised and contrastive learning methods have demonstrated strong potential for learning informative representations under label scarcity; however, their integration with VFL for cross-regional FDIA detection remains largely unexplored.

Motivated by these observations, this work focuses on the less-explored combination of vertical federated learning and self-supervised spatiotemporal representation learning. Specifically, VFedA introduces relational alignment to address the representation inconsistency caused by independently trained regional encoders. Instead of directly forcing heterogeneous feature vectors to match, the proposed mechanism aligns their pairwise similarity structures, allowing the relative relationships among samples to be preserved across regional feature spaces. This design enables cross-regional information fusion while retaining the privacy advantages of federated learning.

VFedA has three key advantages:

- Vertical federated architecture for cross-regional feature fusion. Unlike HFL (same feature space, different samples), VFedA adopts vertical federated learning, in which regions share the same temporal samples but observe different geographical/topological feature spaces. Each regional client extracts local representations through a self-supervised spatiotemporal contrastive learning (STCL) encoder. These representations are concatenated at a control center to form joint global features, enabling the detector to capture system-wide operational characteristics that no single region can observe. At the same time, raw measurements never leave the clients.
- Relational alignment to resolve heterogeneous feature spaces. Independently trained regional encoders produce inconsistent feature spaces. VFedA instead introduces a relational alignment mechanism that aligns pairwise similarity matrices derived from the local representations using a Frobenius norm loss on cosine similarities. This enforces structural consistency across regions without requiring direct feature matching or label information, thereby yielding more stable and discriminative joint representations for the global detector.
- Label efficiency and empirical superiority. Local representation learning is fully self-supervised. No attack labels are required, and only a small fraction of labeled samples (e.g., 1%) is used at the control center to train a lightweight detection head. Extensive experiments on the IEEE 30-bus and IEEE 118-bus systems demonstrate that VFedA consistently outperforms conventional HFL baselines (e.g., HFedA) across accuracy, precision, recall, and F1-score while approaching the performance of fully centralized models. Ablation studies further confirm that removing the relational alignment mechanism causes substantial degradation, especially in recall.

## 3. System Model

Below, we introduce some preliminaries for the later derivations.

### 3.1. Power Grid

The power grid is composed of a large number of buses and transmission lines. Its operating state is strictly constrained by the grid’s topological structure in the spatial dimensions. To characterize the electrical connection relationships between buses, we model the power grid as an undirected graph

(1) G=(V,E,H),

where *V* represents the set of buses, *E* represents the set of transmission lines, and $H\in R^{N\times N}$ is the adjacency matrix, in which *N* denotes the number of buses. To ensure that the graph structure closely mirrors the actual grid’s connection patterns, this paper constructs the adjacency matrix *H* based on the bus admittance matrix *B*. The admittance matrix *B* serves as the fundamental matrix for describing electrical connection relationships between buses in power systems; its off-diagonal elements $B_{ij}$ reflect the electrical connection between bus *i* and bus *j*, while the diagonal elements $B_{ii}$ represent the self-admittance of bus *i* (which is typically a non-zero value). When $B_{ij}\neq 0$ (i≠j), it is assumed that there exists a direct electrical connection between bus *i* and bus *j*. Therefore, the adjacency matrix of the undirected graph G is defined as

(2) $$H_{ij}=\left\{\begin{array}{cc} 1, & Y_{ij}\neq 0,\\ 0, & \mathrm{Else} \end{array}\right..$$

In the temporal dimension, the smart grid collects measurement data from multiple sensors at each sampling instant and uses state estimation to calculate the system’s operating state. At sampling instant *t*, let the state characteristics of bus *i* be represented by $x_{i}\left(t\right)\in R^{F}$, where F denotes the number of state features for the bus. The state characteristics of all buses can be organized into a feature matrix $X_{t}={\left[x_{1}\left(t\right),x_{2}\left(t\right),\ldots ,x_{N}\left(t\right)\right]}^{T}\in R^{N\times F}$. Accordingly, the operating state of the power grid at sampling instant *t* can be expressed as

(3) $$G_{i}=\left(V,E,H;X_{i}\right).$$

Furthermore, considering that the state estimation results obtained at a single moment are often insufficient to fully capture the dynamic nature of the power grid’s operating state, this paper employs a sliding time window to aggregate the operating states of consecutive *l* sampling moments into a sequential sample:

(4) $$S_{t}=\left\{G_{t-l+1},G_{t-l+2},\ldots ,G_{t}\right\}.$$

This sample shares the same topological structure in space while capturing the dynamic evolution of the system’s state over time.

### 3.2. False Data Injection Attack

#### 3.2.1. State Estimation

State estimation is one of the key technologies for ensuring the stable operation of power systems. Its goal is to derive optimal estimates of current network state variables from real-time measurement data and physical models, thereby providing reliable support for dispatch decisions. Due to factors such as equipment accuracy and environmental interference, there are deviations between measured values and true values. State estimation minimizes measurement errors and estimates the system’s state variables while satisfying the physical constraints of the power grid.

In a general form, let the state variables of the system at a given time be $x={\left[x_{1},x_{2},\ldots ,x_{n}\right]}^{T}$, the measured values be $z={\left[z_{1},z_{2},\ldots ,z_{m}\right]}^{T}$, and the measurement error be $n=[n_{1},n_{2},\ldots ,n_{m}]$, where *n* represents the dimension of the state variables and *m* represents the dimension of the measured values collected by the SCADA system. Accordingly, the relationship between the system state and the measurement data can be expressed as

(5) z=h(x)+n,

where $h\left(\cdot \right)={\left[h_{1}\left(\cdot \right),h_{2}\left(\cdot \right),\ldots ,h_{m}\left(\cdot \right)\right]}^{T}$ denotes the functional relationship between state variables and measured values. Under the aforementioned relational constraints, the fundamental task of state estimation is to solve for the optimal estimate of the system state variables $x\in R^{n}$ based on the measured data $z\in R^{m}$. In practical applications, state estimation typically employs optimization methods such as weighted least squares (WLS), which minimizes the weighted sum of squared measurement residuals to obtain the optimal estimate of the system state.

Given that transmission grids play a crucial role in large-scale power transmission, this paper focuses on AC state estimation in transmission grids. In this scenario, the system’s state variables are the voltage magnitude $V_{i}$ and phase angle $\delta_{i}$ at bus *i*, while the measured data include the active power injection $P_{i}$ and reactive power injection $Q_{i}$ at bus *i*. The measurement function h(·) can be expressed as

(6) $$\begin{array}{cc} \; & P_{i}=\sum_{j=1}^{N}V_{i}V_{j}b_{ij}\cos\left(\delta_{ij}\right)+V_{i}V_{j}g_{ij}\sin\left(\delta_{ij}\right),\\ \; & Q_{i}=\sum_{j=1}^{N}V_{i}V_{j}b_{ij}\sin\left(\delta_{ij}\right)-V_{i}V_{j}g_{ij}\cos\left(\delta_{ij}\right), \end{array}$$

where $\delta_{ij}=\delta_{i}-\delta_{j}$ represents the phase angle difference between buses *i* and *j*, $b_{ij}$ and $g_{ij}$ denote the conductance and susceptance between buses *i* and *j*, respectively, and *N* represents the total number of buses in the system.

#### 3.2.2. Bad Data Detection

In power grid state estimation, measurement data collected by SCADA systems may be affected by factors such as equipment failures, communication interference, or malicious attacks, leading to abnormal data that deviate from true values. To ensure the reliability of state estimation results, power systems typically employ a bad data detection (BDD) mechanism to identify anomalous measurement data. The core of this mechanism lies in the fact that normal measurement values satisfy the physical equations constraints with the system’s actual state, whereas abnormal measurement values often exhibit significant deviations.

Let the estimated values of the system’s state variables be denoted by $\hat{x}$. Then, the residual between the actual measured values and the estimated measured values is defined as

(7) $$r=z-h\left(\hat{x}\right).$$

Currently widely adopted bad data detection methods essentially work by determining whether the two-norm of the residual, ${∥r∥}_{2}$, exceeds a pre-set threshold ζ, which is then used to detect whether the measurement value vector z contains any abnormal data. With

(8) $$\left.\left\{\begin{array}{cc} {∥r∥}_{2}\leq \zeta , & \mathrm{Normal}\;\mathrm{measurements},\\ {∥r∥}_{2}>\zeta , & \mathrm{Abnormal}\;\mathrm{measurements}, \end{array}\right.\right.$$

selection of the threshold is typically related to the statistical distribution of measurement errors.

#### 3.2.3. False Data Injection Attacks

In smart grids, the physical operation of the power grid is closely linked to the collection, communication, and computational analysis of measurement data, which provide potential vulnerabilities for cyberattacks. While BDD mechanisms based on residuals can identify randomly tampered abnormal measurement values, their detection effectiveness is compromised when abnormal data violate the system’s physical constraints. False data injection attacks (FDIAs) exploit the power grid’s topology and parameter information to construct attack vectors that align with the system’s power flow patterns, bypassing traditional bad data detection mechanisms.

Suppose that an attacker injects an attack vector $a={\left[a_{1},a_{2},\ldots ,a_{m}\right]}^{T}\in R^{m}$. The tampered measurement data can then be expressed as $z_{a}=z+a$. The goal of the FDIA is to shift the state estimation results away from the original estimated values toward ${\hat{x}}_{a}=x+c$, where $c\in R^{n}$ represents the introduced state deviation vector. To bypass the bad data detection mechanism, the attack vector a must satisfy the consistency condition between the measurement data and the state estimation model. In the context of AC state estimation, a typical method for constructing attack vectors is as follows:

(9) $$a=h\left({\hat{x}}_{a}\right)-h\left(\hat{x}\right).$$

Under these construction conditions, the residual of the attacked measurement data can be expressed as follows:

(10) $$∥r_{a}{∥}_{2}={∥z_{a}-h\left({\hat{x}}_{a}\right)∥}_{2}.$$

Substituting the definitions of $z_{a}$ and a into the above equation yields the following derivation:

(11) $$\begin{array}{c} ∥r_{a}{∥}_{2}={∥z_{a}-h\left({\hat{x}}_{a}\right)∥}_{2}=∥z-h\left(\hat{x}\right){\;∥}_{2}, \end{array}$$

As can be seen, under the aforementioned attack construction method, the residual of the measurement data remains consistent before and after the attack, i.e., $∥r_{a}{∥}_{2}={∥r∥}_{2}$. Since bad data detection mechanisms rely solely on the magnitude of the residual for identification, when the original measurement data meet the detection threshold requirements, the attacked measurement data can also pass detection, giving the FDIA strong stealth capabilities.

We consider a stealthy false data injection adversary that can compromise a subset of measurement channels and modify the corresponding measurement values before they are delivered to the grid monitoring/state estimation system. The attacker is assumed to possess sufficient knowledge of the target grid topology and measurement model to construct physically consistent attack vectors, but does not control the federated learning protocol, regional encoders, or the central detection model. The attack may affect measurements belonging to one or more regions, while the attacker does not necessarily have access to all measurement channels simultaneously. The injected vector *a* is added to the legitimate measurement vector *z*, producing $z^{a}=z+a$, and is constructed according to $a=h\left({\hat{x}}^{a}\right)-h\left(\hat{x}\right)$ so that the resulting measurements remain consistent with the state estimation model and can evade conventional residual-based bad data detection. The objective of the attacker is to induce a non-negligible deviation in the estimated system state while remaining stealthy. Under this threat model, VFedA focuses on detecting compromised temporal samples from distributed regional measurements rather than on identifying the attacker or modifying the federated learning infrastructure.

### 3.3. FDIA Detection

We formulate the FDIA detection task as a binary classification problem. For each temporal sample $S_{t}$, we define its label $y_{t}\in \left\{0,1\right\}$, where $y_{t}=1$ indicates that the sample has been compromised by a false data injection attack and $y_{t}=0$ corresponds to normal operation. The complete sample set is accordingly denoted as $O={\left\{\left(S_{t},y_{t}\right)\right\}}_{t=1}^{N}$, where *N* is the total number of samples. In practice, obtaining attack labels for power system measurements is extremely challenging due to the stealthy nature of FDIAs and the lack of ground-truth data. Consequently, we assume that only a small fraction of samples are labeled, denoted as $O_{L}\subset O$ with $|O_{L}\left|\ll |O\right|$. The detection model, parameterized by θ, is trained by minimizing a composite objective

(12) $$\min_{\theta }\;\ell \left(\theta ;0,0_{L}\right),$$

where ℓ(·) is a loss function designed to exploit both the large amount of unlabeled data (e.g., through self-supervised learning) and the limited labeled instances (e.g., via supervised fine-tuning). This formulation captures a realistic label scarcity scenario.

It is important to note that the above problem formulation assumes that all samples *O* are centrally available for model training. While this centralized assumption facilitates conventional data-driven detection methods, it becomes impractical in modern multi-regional smart grids, where raw data are distributed across multiple operators and cannot be shared due to privacy and regulatory constraints. This limitation motivates the vertical federated learning framework introduced in the next section, which enables collaborative detection without exposing regional data.

The assumption that the control center possesses a small labeled dataset does not imply that regional clients are incapable of obtaining local labels; rather, it represents a label-scarce system-level supervision scenario in which only a limited number of reliable FDIA labels are available to the central coordinating entity. This setting is motivated by the fact that FDIA labels are difficult and costly to obtain and that reliable identification of system-level attacks may require cross-regional operational information. Regional clients primarily rely on unlabeled measurements for self-supervised representation learning, while the control center uses the limited system-level labels to train the global detection head. If regional clients possess additional heterogeneous labels, VFedA can be extended to incorporate such local supervision; however, this is beyond the scope of the present study.

## 4. Problem Formulation

We consider a multi-region smart grid comprising *K* regions. At sampling time *t*, the operational state of the *k*-th region is represented as $G_{k,t}=\left(V_{k},E_{k},H_{k},X_{k,t}\right)$, where $V_{k}$, $E_{k}$, and $H_{k}$ denote the regional bus set, transmission lines, and adjacency matrix, respectively, and $X_{k,t}$ is the node feature matrix. To capture temporal dynamics, a sliding window of length *l* is applied to construct a sequential sample $S_{k},t=\left\{G_{k,t-l+1},\ldots ,G_{k,t}\right\}$. Samples from different regions are concatenated along the regional dimension to form a joint global sample:

$$S_{t}=\left[S_{1,t},S_{2,t},\ldots ,S_{K,t}\right].$$

In this vertical federated setting, each region *k* maintains its own unlabeled dataset $O_{k}=\left\{S_{k,t}\right\}$. The control center possesses only a small labeled dataset $O_{L}\subset O$, where $O=\{S_{t}\}$ and $|O_{L}\left|\ll |O\right|$. Training proceeds in communication rounds. In each round *r*, each region independently updates its local encoder via contrastive learning on $O_{k}$, yielding parameters $\theta_{k}^{r}=$$\arg\min_{\theta }\ell_{cl}\left(\theta ;O_{k}\right)$. The corresponding local representations $z_{k,t}=f_{\theta_{k}}\left(S_{k,t}\right)$ are then uploaded to the control center.

Because the feature spaces of independently trained regional encoders are inherently different, directly concatenating the uploaded representations as $z_{t}=$ concat$\left(z_{1,t},\ldots ,z_{K,t}\right)$ introduces feature distribution mismatches that degrade the discriminative power of the global detector. To overcome this, we introduce a relational alignment constraint $\ell_{\mathrm{al}}\left(\left\{\theta_{k}\right\};O\right)$, which enforces structural consistency across regions by aligning the pairwise similarity structures derived from their respective representations. The control center computes the gradients of this alignment loss with respect to each region’s representations and sends them back to the corresponding clients, enabling the local encoders to incorporate global structural information in subsequent updates. The overall optimization objective for the local encoders is formulated as follows:

(13) $$\min_{\left\{\theta_{k}\right\}}\sum_{k=1}^{K}\ell_{\mathrm{cl}}\left(\theta_{k};O_{k}\right)+\ell_{\mathrm{al}}\left(\left\{\theta_{k}\right\};O\right)$$

where the first term preserves the quality of local representations and the second ensures cross-regional representational compatibility. The global detection model $g_{\phi }$ is trained exclusively at the control center using the concatenated joint representations and the limited labeled samples $O_{L}$, without affecting the local encoders; this formulation addresses the limited observability challenge inherent in multi-region smart grids while rigorously preserving data privacy.

With regard to regional partitioning and sensitivity, the regional partitioning in VFedA is intended to approximate geographically and operationally distributed grid areas. Each region contains a distinct subset of buses and measurements while observing temporally corresponding system states, naturally forming the vertically partitioned feature spaces required by VFL. In the experiments, the IEEE 30-bus and IEEE 118-bus systems are partitioned into two and four regions, respectively, to represent different levels of regionalization. These configurations are selected as appropriately representative multi-regional scenarios.

## 5. Design of the VFedA Method

This section sequentially presents the overall framework and design of VFedA. Based on the longitudinal federated learning framework, the proposed method first learns sample representations that exhibit global consistency via relational structure constraints, then leverages a small amount of labeled data to achieve FDIA detection.

The vertical partition in VFedA is defined along the regional feature dimension while maintaining temporal sample correspondence, rather than along the sample dimension as in HFL. The term feature fusion refers to the fusion of locally learned representations at the control center, whereas the federated nature of VFedA is established by local raw data isolation and iterative representation/gradient-based collaboration.

### 5.1. Overall Framework

**Figure 1:**
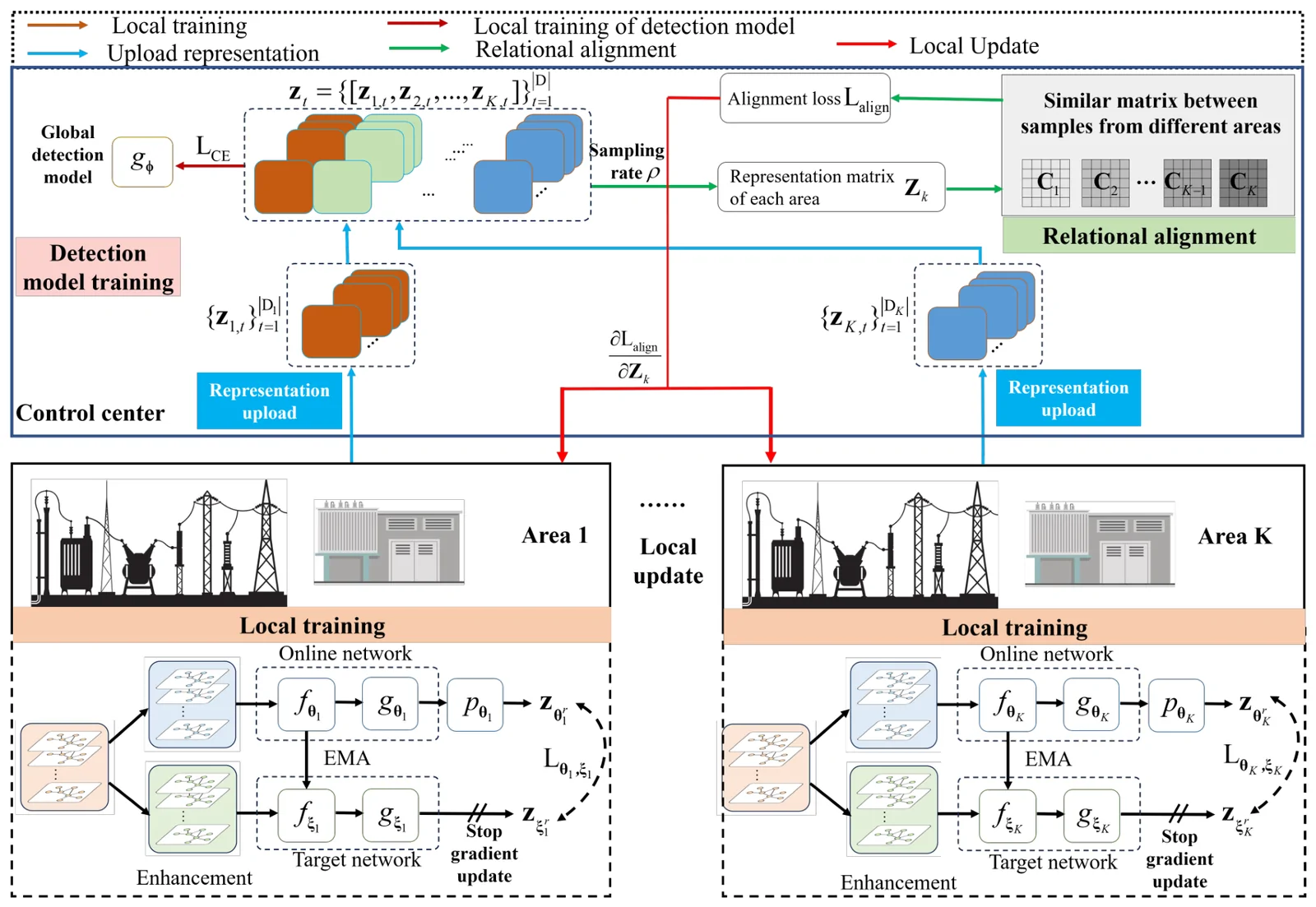
Overview of VFedA.

As shown in Figure 1, the overall training procedure of VFedA proceeds iteratively across a series of communication rounds, comprising five sequential stages that collectively enable collaborative cross-regional feature fusion while preserving data privacy. Each round integrates local representation learning, global aggregation, and structural alignment to progressively refine the encoders and the detection model.

The five stages are detailed as follows:

- Local training: Each regional client independently trains a local encoder using the self-supervised spatiotemporal contrastive learning (STCL) model [4] on its own measurement data. Without requiring any attack labels, this stage learns region-specific representations that capture the underlying operational dynamics of the power grid, thereby establishing a robust feature foundation for subsequent cross-regional collaboration.
- Representation upload: After local training, each client computes the feature representations $z_{k,t}=f_{\theta_{k}}\left(S_{k,t}\right)$ for all samples in its local dataset using the trained encoder. These representations are then transmitted to the central control center, where they are concatenated along the feature dimension to form a unified joint representation $z_{t}=$ concat$\left(z_{1,t},\ldots ,z_{K,t}\right)$. This step effectively aggregates information from all regions, enabling the global model to perceive the holistic operational state of the entire grid.
- Model training for detection: Leveraging the joint representations and a small set of labeled samples available at the control center, a lightweight global FDIA detection model $g_{\phi }\left(\cdot \right)$ is trained using a supervised cross-entropy loss. Importantly, this training phase only updates the parameters of the detection head and does not affect the local encoders, thereby maintaining the privacy and independence of regional feature extraction.
- Relational alignment: To address differences among the feature spaces learned independently by different regions, the control center computes pairwise similarity matrices from the uploaded representations of each region. By minimizing the Frobenius norm difference between these similarity matrices, the relational alignment loss $\ell_{\mathrm{al}}$ enforces structural consistency across regions. This constraint ensures that the relative relationships among samples are preserved in a unified semantic space, which is critical for producing stable and discriminative joint representations.
- Local update: The control center calculates the gradients of the alignment loss with respect to each region’s uploaded representations and transmits these gradient signals back to the corresponding clients. Each client then updates its local encoder parameters via the chain rule, incorporating the cross-region structural information into its own model. This bidirectional interaction closes the communication round and prepares the local encoders for the next iteration of training.

### 5.2. Algorithm Design

We consider a multi-region smart grid comprising *K* regions, each equipped with a local encoder following the contrastive learning architecture proposed in [4]. Let $\theta_{k}$ and $\xi_{k}$ denote the parameters of the online and target networks of region *k*, respectively. The overall training proceeds over *R* communication rounds. In each round, VFedA executes five consecutive phases: local training, representation upload, detection model training, relational alignment, and local update.

#### 5.2.1. Local Training Phase

At the beginning of each round *r*, every region independently performs self-supervised training on its local data using the STCL algorithm [4]. For a given sample $S_{k,t,r}^{r}$, two augmented views are generated and fed into the online network (parameterized by $\theta_{k}^{r}$) and the target network (parameterized by $\xi_{k}^{r}$), producing representations $Z_{k}^{\theta_{r}}$ and $Z_{k}^{\xi_{r}}$, respectively. The local contrastive loss is defined as follows:

(14) $$\ell_{\theta_{k}^{\prime },\xi_{k}^{\prime }}=2-2\cdot \frac{\langle Z_{\theta_{k}^{\prime }},Z_{\xi_{k}^{\prime }}\rangle }{∥Z_{\theta_{k}^{\prime }}{∥}_{2}{∥Z_{\xi_{k}^{\prime }}∥}_{2}}.$$

This objective encourages the encoder to extract stable and informative spatiotemporal features without requiring any labeled data. The target network parameters $\xi_{k}$ are updated via exponential moving average of the online parameters, following standard practice in contrastive learning.

#### 5.2.2. Representation Upload Phase

After local training, each client applies its online encoder $f_{\theta_{k}}\left(\cdot \right)$ to its entire local dataset to obtain feature representations. For any sample $s_{k,t}\in O_{k}$, the extracted representation is

(15) $$z_{k,t}=f_{\theta_{k}}\left(s_{k,t}\right)\in R^{d_{k}},$$

where $d_{k}$ is the feature dimension of region *k*. All clients then upload their respective representations ${\left\{z_{k,t}\right\}}_{t=1}^{|O_{k}|}$ to the control center. The center concatenates the representations from all regions at each time step to form a joint global representation:

(16) $$z_{t}=\mathrm{concat}\left(z_{1,t},z_{2,t},\ldots ,z_{K,t}\right)\in R^{\sum_{k=1}^{K}d_{k}}.$$

This concatenation fuses multi-region information, enabling the subsequent detection model to capture cross-regional operational states that are invisible to any single region.

#### 5.2.3. Detection Model Training Phase

Using the joint representations $z_{t}$ and a small set of labeled samples $O_{L}$ available at the control center, we train a global detection model $g_{\phi }\left(\cdot \right)$ parameterized by ϕ. The model outputs a prediction ${\hat{y}}_{t}=g_{\phi }\left(z_{t}\right)$ for each labeled sample. The training objective is the standard cross-entropy loss:

(17) $$\ell_{\mathrm{TO}}=-\frac{1}{|O_{L}|}\sum_{t\in O_{L}}\left[y_{t}\log\left({\hat{y}}_{t}\right)+\left(1-y_{t}\right)\log\left(1-{\hat{y}}_{t}\right)\right]$$

where $y_{t}$ denotes the ground-truth label associated with the corresponding joint representation $z_{t}$.

Importantly, this supervised loss only updates the parameters ϕ of the detection head, and does not affect the local encoders. This design preserves the privacy of regional data and allows the detection model to exploit the rich cross-region representations without interfering with the unsupervised feature learning process.

#### 5.2.4. Relational Alignment Phase

In vertical federated learning, regional clients observe different feature spaces, so their locally learned representations may vary in their dimensionality, coordinate systems, and feature semantics. Direct feature-level matching may impose an unnecessarily restrictive constraint by forcing heterogeneous representations to become numerically similar. To address this issue, VFedA aligns the relational structures induced by local representations rather than directly matching feature vectors. Specifically, normalized regional representations are used to construct cosine similarity matrices, and the Frobenius distance between the similarity matrices is minimized. This strategy preserves the local coordinate systems and complementary information of individual regions while encouraging corresponding samples to maintain consistent relative relationships across regions. Consequently, the control center can obtain more structurally compatible representations for cross-regional fusion without requiring direct feature correspondence.

Since each region trains its encoder independently, the resulting feature spaces may be inconsistent, which can degrade the quality of the concatenated joint representations. To mitigate this, we introduce a relational alignment mechanism that enforces structural consistency across regions by aligning the pairwise similarity matrices derived from their respective representations. To reduce computational overhead, we randomly sample a subset of indices $L_{\mathrm{all}}$ with size $N_{a}=\rho \left|O\right|$, where ρ is the alignment sampling rate. For region *k*, let $Z_{k}\in R^{N_{a}\times d_{k}}$ be the feature matrix of the sampled samples. We first normalize each row vector to unit length:

(18) $${\tilde{Z}}_{k}=\mathrm{diag}\left(\frac{1}{∥z_{k,1}{∥}_{2}},\ldots ,\frac{1}{∥z_{k,N_{a}}{∥}_{2}}\right)Z_{k}.$$

Then, the cosine similarity matrix for region *k* is computed as follows:

(19) $$D_{k}={\tilde{Z}}_{k}{\tilde{Z}}_{k}^{⊤}$$

where each entry $D_{k}\left(i,j\right)=\frac{\langle z_{k,i},z_{k,j}\rangle }{∥z_{k,i}{∥}_{2}{∥z_{k,j}∥}_{2}}$ reflects the similarity between the *i*-th and *j*-th samples in that region’s feature space. The relational alignment loss is defined as the average Frobenius norm difference between the similarity matrices of all region pairs:

(20) $$\ell_{\mathrm{al}}=\frac{2}{K(K-1)}\sum_{1\leq u<v\leq K}{∥D_{u}-D_{v}∥}_{F}^{2}.$$

Minimizing this loss encourages the relative sample-to-sample relationships to be consistent across regions, thereby aligning the feature spaces into a common semantic structure without requiring direct feature matching.

#### 5.2.5. Local Update Phase

After computing the alignment loss, the control center calculates the gradient of $\ell_{\mathrm{al}}$ with respect to each region’s feature matrix $Z_{k}$. Since $Z_{k}$ depends on the local encoder parameters $\theta_{k,l}^{r}$, the gradient with respect to $\theta_{k}^{r}$ is obtained via the chain rule:

(21) $$\nabla_{\theta_{k}^{r}}\ell_{\mathrm{al}}=\frac{\partial \ell_{\mathrm{al}}}{\partial Z_{k}}\cdot \frac{\partial Z_{k}}{\partial \theta_{k}^{r}}.$$

The control center transmits these gradient signals back to the respective clients. Each client then updates its online encoder parameters using gradient descent:

(22) $$\theta_{k}^{r+1}\leftarrow \theta_{k}^{r}-\eta \nabla_{\theta_{k}^{r}}\ell_{\mathrm{al}}$$

where η is the learning rate. This update incorporates cross-region structural information into the local models, enabling the encoders to gradually produce more compatible representations across all regions. The entire process repeats for *R* communication rounds until convergence. At the inference stage, each client only needs to upload its local representations to the control center, which then applies the trained global detection model $g_{\phi }$ to the concatenated joint representation for real-time FDIA detection. Because raw measurements never leave the regional clients, this architecture maintains data privacy throughout training and deployment.

### 5.3. Convergence Analysis of VFedA

Although the paper does not provide a formal proof of convergence, we present a rigorous justification based on the optimization objectives, following standard non-convex stochastic optimization theory for federated learning.

#### 5.3.1. Global Objective and Problem Structure

Let $\Theta ={\left\{\theta_{k}\right\}}_{k=1}^{K}$ denote the set of local encoder parameters. The overall training objective for the encoders is

(23) $$L\left(\Theta \right)=\sum_{k=1}^{K}E_{S∼O_{k}}\left[\ell_{\mathrm{cl}}\left(\theta_{k};S\right)\right]+\lambda \;\ell_{\mathrm{al}}\left(\Theta \right),$$

where

(24) $$\ell_{\mathrm{al}}\left(\Theta \right)=\frac{2}{K(K-1)}\sum_{1\leq u<v\leq K}{∥D_{u}-D_{v}∥}_{F}^{2},$$

with $D_{k}={\tilde{Z}}_{k}{\tilde{Z}}_{k}^{⊤}$ and ${\tilde{Z}}_{k}$ being the row-normalized feature matrix of region *k*. The detection head $g_{\phi }$ is trained separately by minimizing $\ell_{\mathrm{TO}}$, and does not affect the encoder updates; hence, we focus solely on the convergence of Θ.

#### 5.3.2. Assumptions

We adopt standard assumptions that are implicitly satisfied in the experimental setup.

(A1) Smoothness: Each local loss $\ell_{\mathrm{cl}}\left(\theta_{k}\right)$ and the alignment loss $\ell_{\mathrm{al}}\left(\Theta \right)$ are *Q*-smooth with respect to $\theta_{k}$, i.e., for any $\theta_{k}^{\left(1\right)},\theta_{k}^{\left(2\right)}$, we have

(25) $$∥\nabla_{\theta_{k}}\ell_{\mathrm{cl}}\left(\theta_{k}^{\left(1\right)}\right)-\nabla_{\theta_{k}}\ell_{\mathrm{cl}}\left(\theta_{k}^{\left(2\right)}\right)∥\leq Q∥\theta_{k}^{\left(1\right)}-\theta_{k}^{\left(2\right)}∥,$$

and similarly for $\ell_{\mathrm{al}}$. This is guaranteed by the Lipschitz continuity of the encoder and the cosine similarity.

(A2) Bounded stochastic gradients: During local contrastive learning, the stochastic gradient $g_{k}^{\left(r\right)}$ satisfies

(26) $$E\left[g_{k}^{\left(r\right)}\right]=\nabla_{\theta_{k}}\ell_{\mathrm{cl}}\left(\theta_{k}^{r}\right),\;E[∥g_{k}^{\left(r\right)}-\nabla_{\theta_{k}}\ell_{\mathrm{cl}}\left(\theta_{k}^{r}\right){∥}^{2}]\leq \sigma^{2}.$$

(A3) Unbiased alignment gradient: Sub-sampling of the alignment set $L_{\mathrm{al}}$ provides an unbiased estimator of $\ell_{\mathrm{al}}$, and its gradient variance is bounded by $\sigma_{\mathrm{al}}^{2}/\rho$.

(A4) Bounded parameters: The parameter space is bounded, ensuring that L is lower bounded by some $L^{*}.$

#### 5.3.3. Two-Step Update Scheme

- Local step: For $E_{1}$ epochs, each region updates $\theta_{k}$ by SGD on its own contrastive loss:
  (27) $$\theta_{k}^{\left(r,t+1\right)}=\theta_{k}^{\left(r,t\right)}-\eta \;g_{k}^{\left(r,t\right)},\;t=0,\ldots ,E_{1}-1$$
  with $\theta_{k}^{\left(r,0\right)}=\theta_{k}^{r}$. After these steps, we obtain an intermediate ${\tilde{\theta }}_{k}^{r}$.
- Global alignment step: The control center computes the gradient of $\ell_{\mathrm{al}}$ w.r.t. $\theta_{k}$ (via the chain rule) and sends it back. Then, each region updates
  (28) $$\theta_{k}^{r+1}={\tilde{\theta }}_{k}^{r}-\eta \;\nabla_{\theta_{k}}\ell_{\mathrm{al}}\left({\tilde{\Theta }}^{r}\right).$$
  This is an alternating descent on a composite objective.

#### 5.3.4. Convergence Rate for Non-Convex Objectives

We treat the whole process as a single stochastic gradient descent on L with a biased gradient estimator. However, we can decouple the effects using the following lemma.

**Lemma** **1.** *Under (A1)–(A4), if the learning rate η≤1/(2Q), then*

(29) $$E\left[L\left(\Theta^{r+1}\right)\right]\leq E\left[L\left(\Theta^{r}\right)\right]-\frac{\eta }{2}\sum_{k=1}^{K}E[∥\nabla_{\theta_{k}}L\left(\Theta^{r}\right){∥}^{2}]+\frac{\eta Q}{2}\left(\sigma^{2}+\frac{\sigma_{\mathrm{al}}^{2}}{\rho }\right)+O\left(\eta^{2}Q^{2}E_{1}^{2}\right).$$

**Theorem** **1.** *Under the assumptions of Lemma 1, after R communication rounds,*

(30) $$\min_{0\leq r\leq R-1}E\left[∥\nabla L\left(\Theta^{r}\right){∥}^{2}\right]\leq \frac{2(L\left(\Theta^{0}\right)-L^{*})}{R\eta }+\frac{\eta Q}{2}\left(\sigma^{2}+\frac{\sigma_{\mathrm{al}}^{2}}{\rho }\right)+O\left(\frac{1}{E_{1}}\right).$$

**Proof.**  Starting from Lemma 1, we sum the inequality over r=0,1,…,R−1:

(31) $$\sum_{r=0}^{R-1}E\left[L\left(\Theta^{r+1}\right)-L\left(\Theta^{r}\right)\right]\leq -\frac{\eta }{2}\sum_{r=0}^{R-1}\sum_{k=1}^{K}E[∥\nabla_{\theta_{k}}L\left(\Theta^{r}\right){∥}^{2}]+\frac{R\eta Q}{2}\left(\sigma^{2}+\frac{\sigma_{\mathrm{al}}^{2}}{\rho }\right)+R\cdot O\left(\eta^{2}Q^{2}\delta^{2}\right).$$

The left-hand side is a telescoping sum:

(32) $$\sum_{r=0}^{R-1}E\left[L\left(\Theta^{r+1}\right)-L\left(\Theta^{r}\right)\right]=E\left[L\left(\Theta^{R}\right)\right]-L\left(\Theta^{0}\right).$$

Substituting this and rearranging terms to isolate the gradient norm sum,

(33) $$\frac{\eta }{2}\sum_{r=0}^{R-1}E[∥\nabla L\left(\Theta^{r}\right){∥}^{2}]\leq L\left(\Theta^{0}\right)-E\left[L\left(\Theta^{R}\right)\right]+\frac{R\eta Q}{2}\left(\sigma^{2}+\frac{\sigma_{\mathrm{al}}^{2}}{\rho }\right)+R\cdot O\left(\eta^{2}Q^{2}E_{1}\sigma^{2}\right).$$

Since L is bounded below by $L^{*}$,we have $-E\left[L\left(\Theta^{R}\right)\right]\leq -L^{*}$. Hence,

(34) $$\frac{\eta }{2}\sum_{r=0}^{R-1}E[∥\nabla L\left(\Theta^{r}\right){∥}^{2}]\leq L\left(\Theta^{0}\right)-L^{*}+\frac{R\eta Q}{2}\left(\sigma^{2}+\frac{\sigma_{\mathrm{al}}^{2}}{\rho }\right)+R\cdot O\left(\eta^{2}Q^{2}E_{1}\sigma^{2}\right).$$

Now, multiply both sides by $\frac{2}{R\eta }:$

(35) $$\frac{1}{R}\sum_{r=0}^{R-1}E[∥\nabla L\left(\Theta^{r}\right){∥}^{2}]\leq \frac{2(L\left(\Theta^{0}\right)-L^{*})}{R\eta }+\frac{\eta Q}{2}\left(\sigma^{2}+\frac{\sigma_{\mathrm{al}}^{2}}{\rho }\right)+O\left(\eta^{2}Q^{2}E_{1}\sigma^{2}\right).$$

Finally, the average over *R* rounds is at least the minimum over those rounds:

(36) $$\min_{0\leq r\leq R-1}E[∥\nabla L\left(\Theta^{r}\right){∥}^{2}]\leq \frac{1}{R}\sum_{r=0}^{R-1}E[∥\nabla L\left(\Theta^{r}\right){∥}^{2}].$$

Therefore, we have

(37) $$\min_{0\leq r\leq R-1}E[∥\nabla L\left(\Theta^{r}\right){∥}^{2}]\leq \frac{2(L\left(\Theta^{0}\right)-L^{*})}{R\eta }+\frac{\eta Q}{2}\left(\sigma^{2}+\frac{\sigma_{\mathrm{al}}^{2}}{\rho }\right)+O\left(\eta^{2}Q^{2}E_{1}\sigma^{2}\right).$$

By choosing the learning rate $\eta =O(1/\sqrt{R})$, the first term decays as $O(1/\sqrt{R})$ and the second term also decays as $O(1/\sqrt{R})$. The term $O(\eta^{2}Q^{2}E_{1}\sigma^{2})$ becomes O(1/R), which is of lower order. Thus, the overall convergence rate is

(38) $$\min_{0\leq r\leq R-1}E[∥\nabla L\left(\Theta^{r}\right){∥}^{2}]=O\left(\frac{1}{\sqrt{R}}\right)+O\left(\frac{1}{E_{1}}\right).$$

This completes the proof of Theorem 1.

□

### 5.4. Communication and Scalability Analysis

VFedA does not transmit raw regional measurements; instead, communication mainly consists of local representation uploading and alignment-gradient feedback. Given *K* regions, *N* aligned samples, and regional representation dimensions $d_{k}$, the representation upload cost per communication round is proportional to $\sum_{k=1}^{K}Nd_{k}$. To control the computational cost of relational alignment, only $N_{a}=\rho \left|O\right|$ samples are randomly selected, and the similarity matrix for each region is computed with complexity approximately $O(N_{a}^{2}d_{k})$. The pairwise relational loss introduces an additional dependence on the number of regions, approximately $O(K^{2}N_{a}^{2})$. Therefore, VFedA achieves scalability by combining compact representation exchange with alignment sampling, although the quadratic dependence on the number of regions represents an important limitation for extremely large regional deployments.

## 6. Experimental Evaluation

To verify the effectiveness of the VFedA method in detecting FDIAs, this section conducts experimental evaluations on power systems of varying scales.

### 6.1. Experimental Setup

#### 6.1.1. Dataset Setup

We employ the dataset from the California Independent System Operator (CAISO) (CAISO load data, https://www.caiso.com/generation-transmission/load, accessed on 1 July 2026). Real 2019 load data were used, and a power grid operation simulation environment was established in MATLAB 2019b on both the IEEE 30-bus and IEEE 118-bus standard systems. Power flow calculations and state estimation were performed using the MATLAB Power System Toolbox (MATPOWER), with a 5-min time resolution for state estimation.

Attack samples were constructed through the aforementioned process. The IEEE 30-bus and IEEE 118-bus systems were divided into two regions and four regions, respectively. Each region contained 10,000 continuous-time samples and was split into training and test sets in an 8:2 ratio. The training set was used for federated contrastive learning and model training, while the test set was used solely for performance evaluation.

#### 6.1.2. Baseline Methods and Comparison Methods

To evaluate the performance of the proposed VFedA method for FDIA detection tasks, this paper selects two training modes as baselines and compares them with existing longitudinal federated learning approaches. Among the baseline methods, we primarily include centralized detection methods (STCL) [29] and HFL methods (HFedA) [4]. For comparison methods, given that research on longitudinal federated learning for FDIA detection remains relatively limited, we selected the most representative methods from existing studies. VFL-FDIA [10] uses an FDIA detection model based on longitudinal federated learning; each participant extracts spatial features from the grid operation data locally and uploads them to the server, then the server uses a Bi-LSTM to model temporal features and complete the detection process. To ensure fairness in the comparison experiments, all methods followed the model architecture and training strategies described in the original papers and were implemented under unified experimental conditions.

#### 6.1.3. Training Settings

This paper implements the proposed VFedA method in PyTorch v2.10. Experiments were conducted on hardware equipped with an Intel i9-14900HX CPU (Santa Clara, CA, USA), an NVIDIA GTX 4060 GPU (Santa Clara, CA, USA), and 32 GB of memory. During federated training, the number of communication rounds was set to R=20. Each round involved five local training iterations, with a momentum coefficient of τ=0.99 for contrastive learning. The detection model was trained over 50 rounds using 1% of the labeled samples. The learning rate was set to η=0.0002, the batch size to 32, and the alignment sampling rate to ρ=0.1. The sliding time window length was set to l=3, and the number of attention heads in GAT was set to M=6.

#### 6.1.4. Evaluation Metrics

Our experiments employ accuracy, recall, precision, and F1 score as the evaluation metrics. Given that the number of normal samples versus attack samples is typically imbalanced in FDIA scenarios, the recall, precision, and F1 score are calculated using a macro-average approach. It is worth noting that in vertical federated learning scenarios, unlike HFL, all evaluation metrics are computed for the global detection model.

### 6.2. Experimental Results

#### 6.2.1. Comparison Results

**Figure 2:**
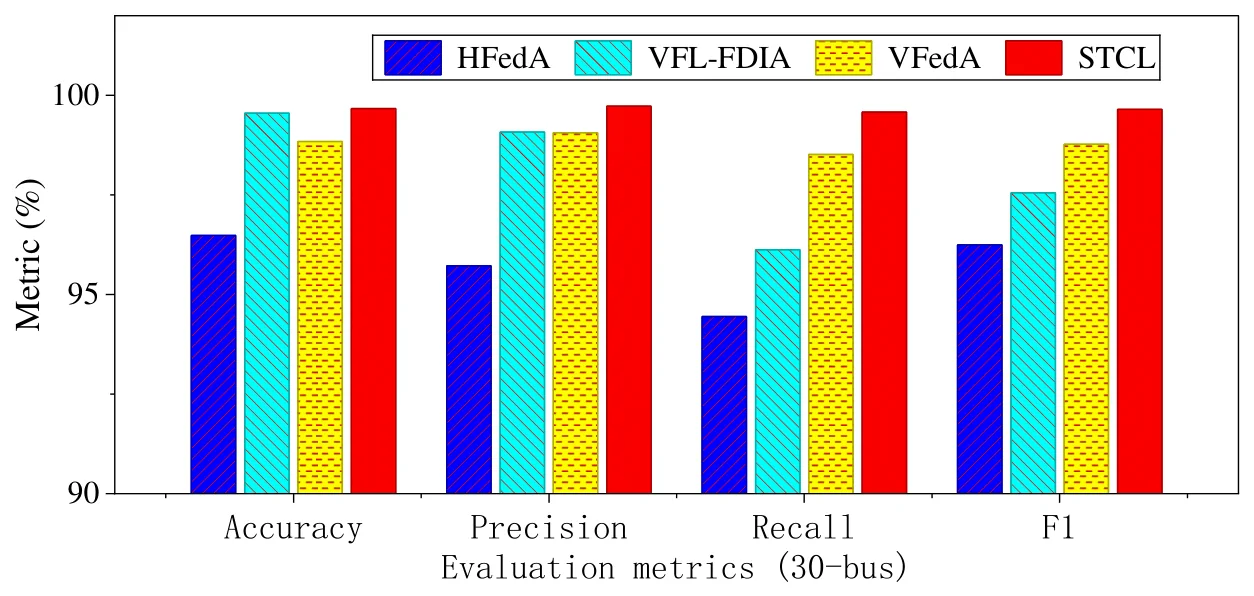
FDIA detection performance of different methods with the IEEE 30-bus power system.

We first evaluate the FDIA detection performance of all competing methods on the IEEE 30-bus system, with the results presented in Figure 2. VFedA achieves superior detection performance across all evaluation metrics, attaining accuracy of 98.84%, precision of 99.06%, recall of 98.52%, and F1 score of 98.77%. Compared with VFL-FDIA, which operates under a supervised learning paradigm, VFedA demonstrates highly competitive overall performance while requiring substantially fewer labeled samples. Notably, VFedA exhibits slight improvements in recall and F1 score, suggesting that the proposed self-supervised approach with relational alignment is particularly effective at identifying attack instances even under label-scarce conditions.

As shown in Figure 2, VFedA substantially outperforms HFedA across all four evaluation metrics, with particularly pronounced gains in recall and F1 score. This performance gap can be attributed to the fundamental limitation of HFL approaches, which rely exclusively on local measurement data and lack access to cross-regional operational characteristics. In contrast, VFedA’s vertical federated learning framework enables comprehensive cross-regional information fusion, effectively mitigating performance degradation due to limited regional observability.

**Figure 3:**
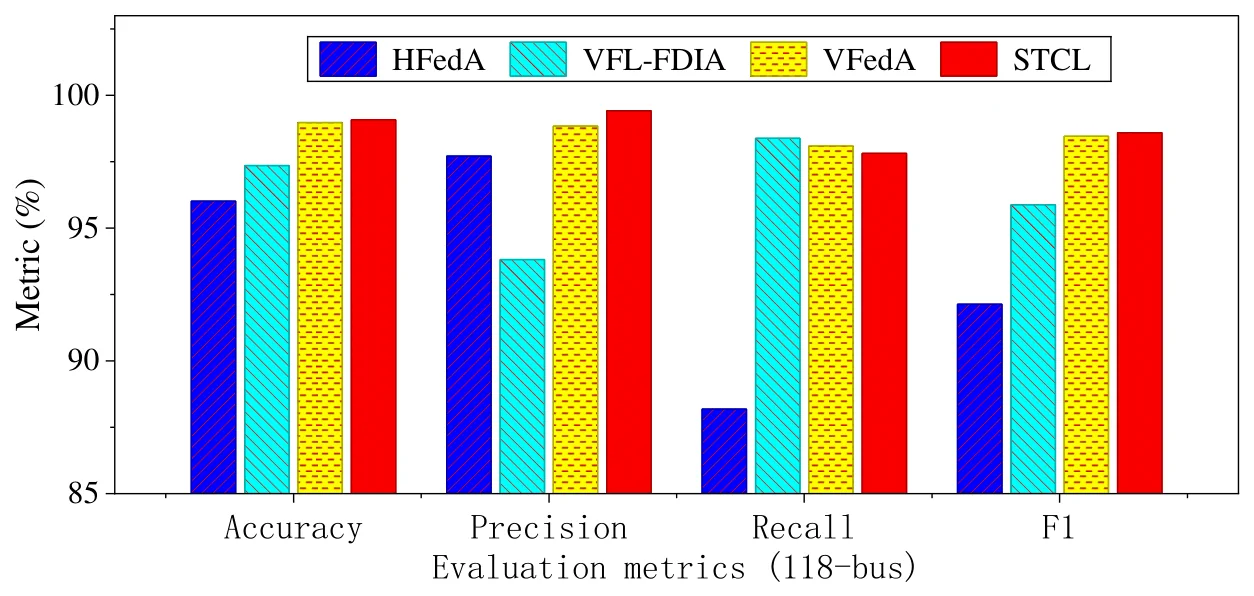
FDIA detection performance of different methods with the IEEE 118-bus power system.

We conducted the same comparative evaluation on the IEEE 118-bus system, with the results illustrated in Figure 3. The increased system scale presents greater challenges for FDIA detection, imposing higher demands on model capacity and generalization. Under these more demanding conditions, both vertical federated learning methods demonstrate strong stability and maintain high performance levels. VFedA achieves accuracy of 98.98%, precision of 98.84%, recall of 98.09%, and an F1 score of 98.46%, attaining optimal or near-optimal results across all evaluation metrics.

Notably, performance disparities among methods become more pronounced as the system scale increases. HFedA experiences the most significant performance degradation, with recall dropping to 88.18%, indicating that local measurement data alone are insufficient to capture the full operational characteristics across multiple regions and result in substantially higher false negative rates. In contrast, VFedA maintains consistently high performance across all metrics thanks to its integration of multi-region representation information, with only a modest gap relative to centralized detection methods. This demonstrates the robustness and scalability of the proposed approach in complex large-scale grid environments.

#### 6.2.2. Ablation Study

**Figure 4:**
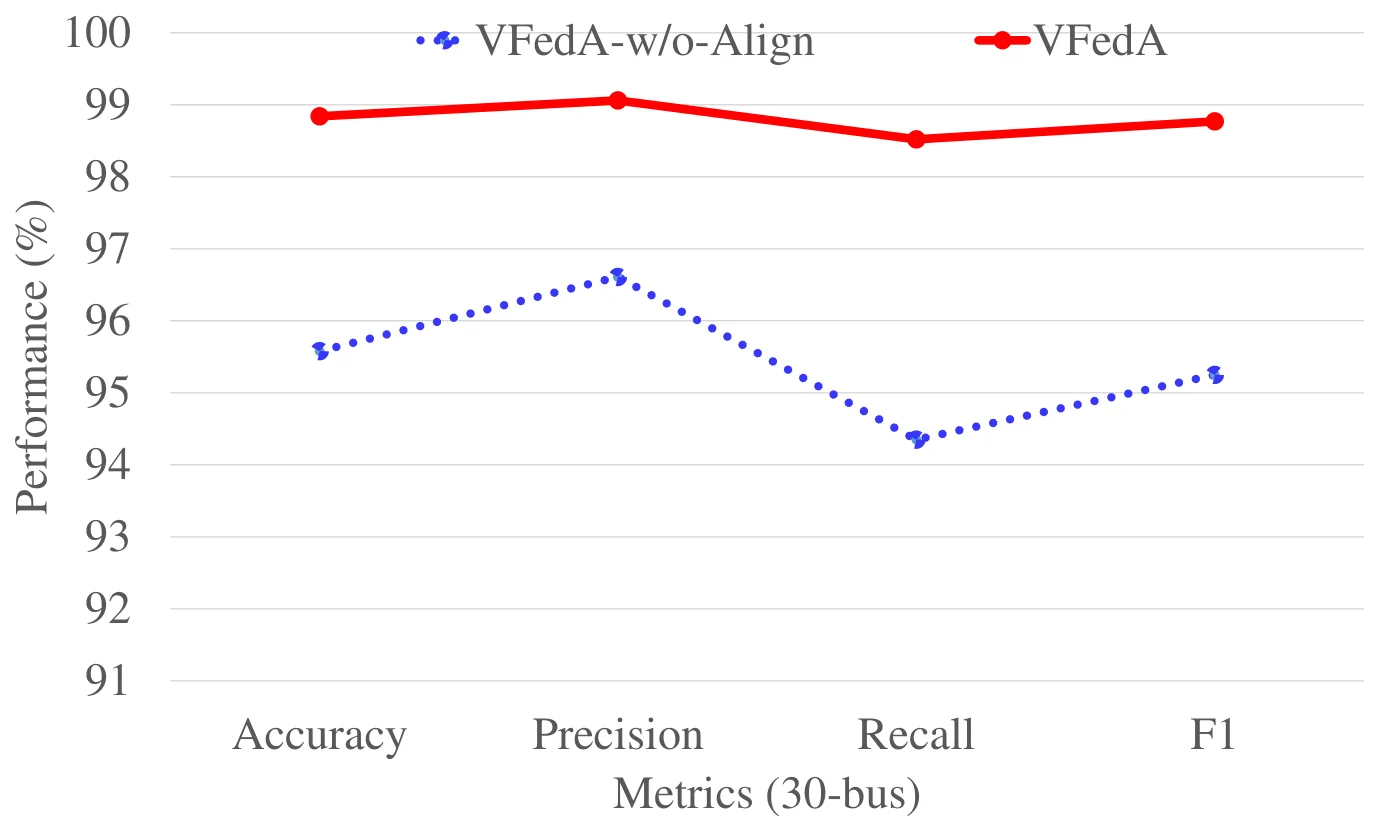
Ablation study results for the alignment mechanism with the IEEE 30-bus power system.

**Figure 5:**
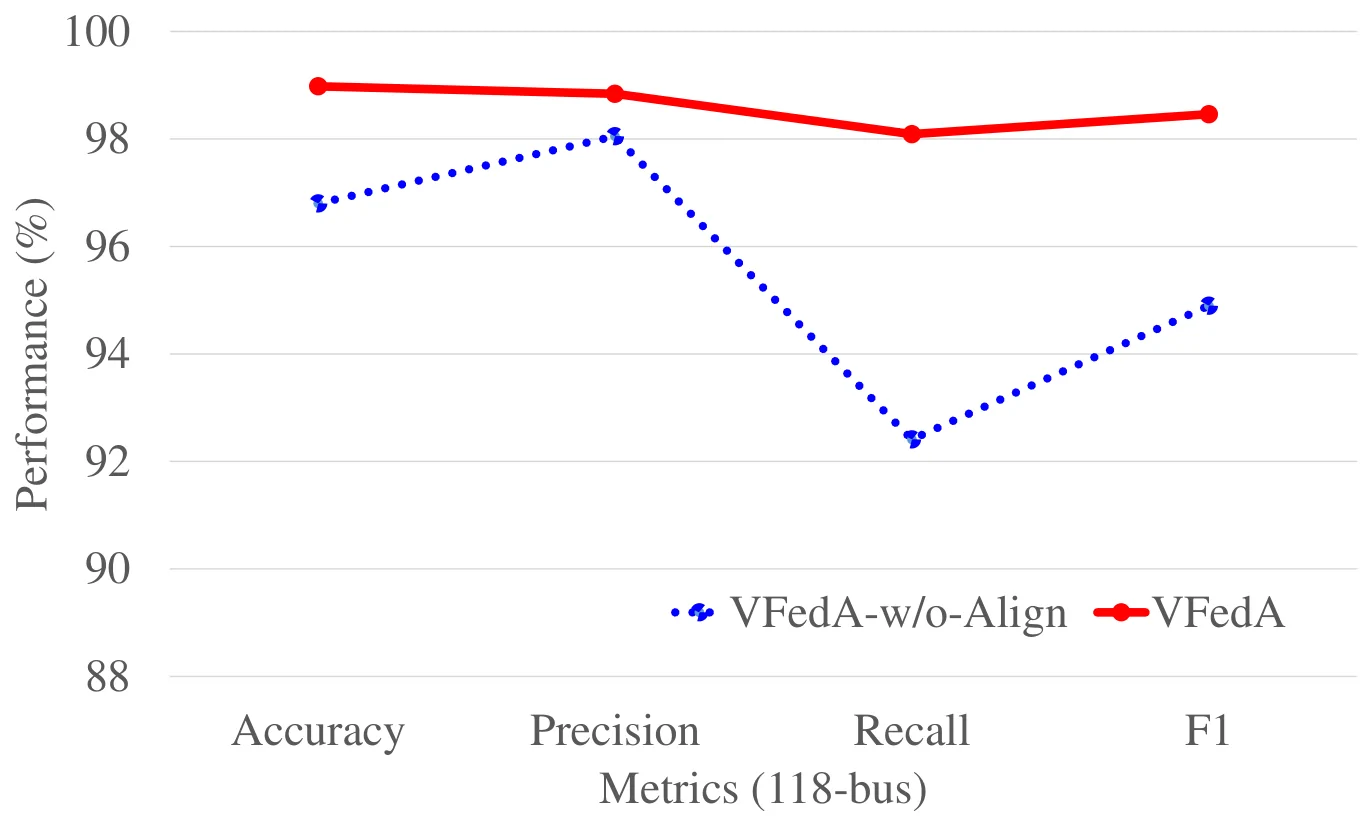
Ablation study results for the alignment mechanism with the IEEE 118-bus power system.

To rigorously validate the effectiveness of the key components in VFedA, we conducted ablation experiments on both the IEEE 30-bus and IEEE 118-bus systems, comparing the complete model against a variant that removes the relational alignment mechanism (VFedA-w/o-Align). As shown in Figure 4 and Figure 5, the complete VFedA method consistently outperforms VFedA-w/o-Align across all evaluation metrics on both test systems.

On the IEEE 30-bus system, removing the alignment mechanism leads to substantial performance degradation: accuracy drops to 95.58% (a decline of 3.26 percentage points), precision falls to 96.61% (a decline of 2.45 percentage points), recall decreases to 94.35% (a decline of 4.17 percentage points), and F1 score falls to 95.25% (a decline of 3.52 percentage points). Similarly, on the IEEE 118-bus system, the recall metric drops significantly to 92.41% after removing the alignment mechanism, underscoring the critical role of relational alignment in enhancing detection capabilities.

The performance gap is particularly pronounced for the recall metric across both test systems, indicating that the alignment mechanism effectively reduces false negatives in attack detection. The primary reason for this performance disparity lies in the inherent distributional differences in measurement data collected across regions within a vertical federated learning framework. Without effective feature alignment, representations from distinct regions fail to form a consistent and coherent representation space at the control center, hindering the global model’s ability to leverage cross-region information. When introducing the relational alignment mechanism, feature representations from different regions are better aligned in the feature space, which substantially enhances the model’s capacity for cross-regional information fusion and improves its overall detection performance.

#### 6.2.3. Communication Round and Parameter Sensitivity Analysis

**Figure 6:**
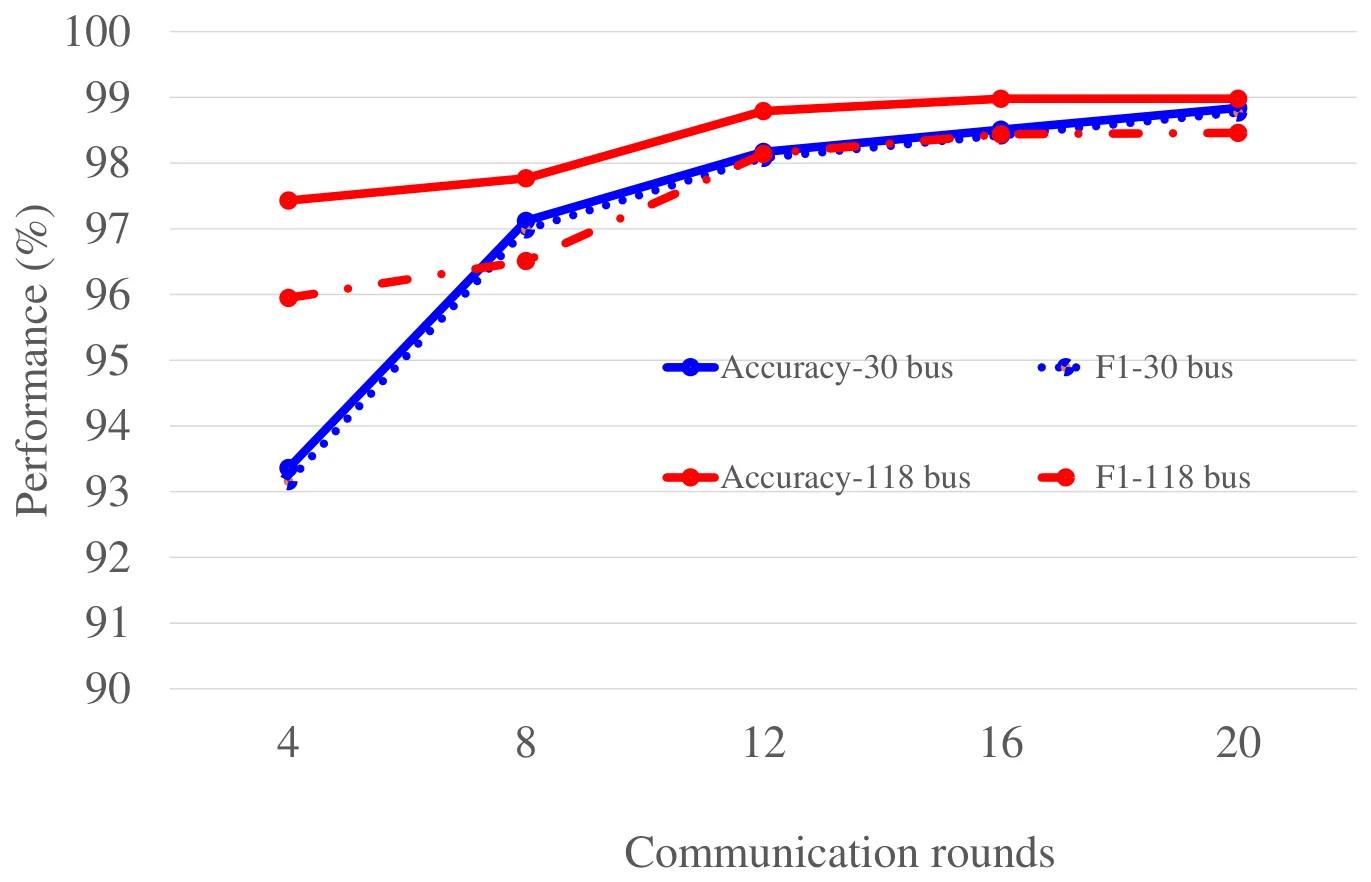
Performance of VFedA for different communication rounds with the IEEE 30-bus power system.

In federated learning frameworks, the number of communication rounds directly influences the degree of collaboration between the control center and regional clients, thereby affecting the model’s detection performance. To analyze VFedA’s behavior under varying communication rounds, we conducted experiments on both the IEEE 30-bus and IEEE 118-bus systems. As shown in Figure 6, both accuracy and F1 score increase progressively as the number of communication rounds increases. Performance gains are most rapid in the initial stages, with metrics improving substantially from rounds 4 to 12. The rate of improvement begins to decelerate beyond 12 rounds, with performance stabilizing at around 20 rounds. This trend indicates that as the number of communication rounds increase, clients progressively share cross-region information and iteratively optimize the global model, thereby enhancing FDIA detection performance until convergence.

**Figure 7:**
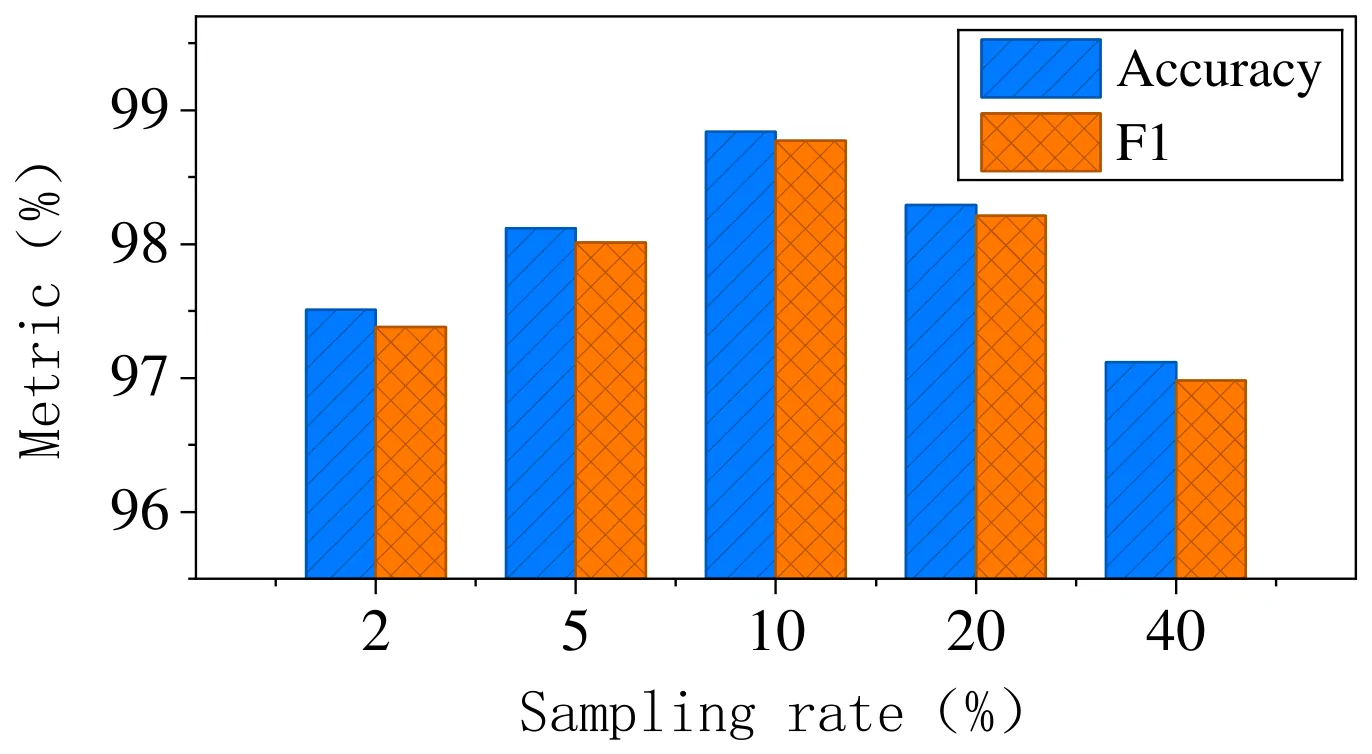
Performance of VFedA for different sampling rates with the IEEE 30-bus power system.

We further analyze the impact of the alignment sampling rate ρ on detection performance, with results depicted in Figure 7. As the sampling rate increases from 2% to 10%, both accuracy and F1 score demonstrate steady improvement. This can be attributed to higher sampling rates providing more comprehensive pairwise relationships for alignment, enabling more robust structural consistency across regional feature spaces. However, performance gains become marginal beyond 10%, while computational overhead increases substantially. This suggests that the selected sampling rate of ρ=0.1 effectively balances detection performance and computational efficiency, providing near-optimal alignment benefits without incurring prohibitive computational costs. The sensitivity analysis confirms that VFedA maintains stable performance across a range of hyperparameter settings, demonstrating its practical applicability in real-world deployment scenarios.

In a federated learning framework, the number of communication rounds directly influences the degree of collaboration between the control center and client devices in each region, in turn affecting the model’s detection performance. To analyze how VFedA performs under varying numbers of communication rounds, we conducted further experiments on both the IEEE-30-bus and IEEE-118-bus systems. As shown in Figure 6, both accuracy and F1 score gradually improve as the number of communication rounds increases. The rate of improvement in each metric begins to slow once the number of communication rounds increases past 12, eventually stabilizing after around 20 rounds. These results indicate that clients can progressively share cross-region information to optimize the global model as the number of communication rounds increases, leading to improved FDIA detection performance.

### 6.3. Discussion of the Experimental Results

Although the experiments in this paper were conducted on IEEE 30-bus and IEEE 118-bus benchmark systems, the operating conditions were driven by real 2019 CAISO load data. These data have a 5-min temporal resolution and do not include synthetic load trajectories. Moreover, the networks are explicitly partitioned into multiple geographical regions. Two regions were used for IEEE 30-bus system and four for the IEEE 118-bus system. This partitioning reproduces the heterogeneous and distributed observability characteristics targeted by the vertical federated learning setting. The experimental results on two network scales further demonstrate that VFedA can effectively exploit complementary cross-regional information. Although VFedA maintains robust detection performance under increasing system complexity, the current study does not include synchronized real-world multi-regional measurement datasets from operational power grids, which would allow for a more comprehensive evaluation under realistic sensor noise, missing measurements, communication imperfections, topology changes, and varying measurement configurations. We consider validation using multi-source real-world SCADA/PMU measurements and more diverse regional operating conditions to be an important direction for future work.

The contribution of VFedA is not solely in the application of federated learning or contrastive representation learning to FDIA detection; its main methodological contribution is the integration of vertical cross-regional representation learning with relational alignment. Existing HFL-based approaches primarily aggregate knowledge across clients with distinct sample spaces, while existing vertical approaches generally do not address the semantic inconsistency among independently learned regional representation spaces. VFedA establishes sample-level correspondence across regions, learns regional representations without relying heavily on attack labels, and uses relational alignment to make differing regional representations structurally compatible prior to global detection.

VFL-FDIA mainly addresses how spatial and temporal information can be collaboratively processed under VFL. VFedA additionally addresses how heterogeneous regional representations can be coordinated at the relational level; in this way, the proposed relational alignment mechanism complements the VFL architecture while preserving cross-regional sample relationships. This is particularly important for FDIA detection scenarios involving limited regional observability and scarce labeled attack data.

## 7. Discussion

VFedA prevents the disclosure of raw regional measurements by performing representation learning locally; however, the uploaded latent representations and relational similarity matrices may still contain information correlated with the underlying grid operating states. In particular, the representation $z_{k,t}$ preserves spatiotemporal characteristics required for FDIA detection, while the similarity matrix $D_{k}$ reveals pairwise relationships among regional samples. Therefore, our privacy claims in this paper apply to the privacy of raw data, not to protection against representation inference or reconstruction attacks. The proposed framework does not currently provide differential privacy or cryptographic protection for intermediate representations. A more comprehensive privacy analysis covering representation reconstruction, membership inference, and attribute inference attacks will be investigated in future work. Privacy-enhancing mechanisms such as differential privacy or secure computation should be considered as well.

VFedA achieves detection performance close to centralized STCL; however, its practical advantages need to be evaluated jointly from the perspectives of detection effectiveness, communication overhead, computational cost, and privacy. Unlike centralized detection, VFedA keeps regional raw measurements local; it exchanges learned representations and alignment gradients with the control center. Compared with conventional HFL, VFedA incurs additional representation-level communication and relational alignment computation, but obtains substantially richer cross-regional information; consequently, it improves FDIA detection even under limited regional observability. The alignment sampling rate and communication-round analyses further demonstrate a trade-off between collaborative information utilization and computational/communication overhead. VFedA seeks to approach the performance of centralized detection without requiring centralized access to raw regional measurements, and as such should be viewed as a compromise between the goals of privacy, accuracy, and efficiency.

## 8. Conclusions

This paper proposes VFedA, a vertical federated learning framework with relational alignment for detecting false data injection attacks in multi-regional smart grids. Local clients extract spatiotemporal features via self-supervised contrastive learning, then upload them to a central controller for global fusion. A relational alignment mechanism mitigates feature space inconsistencies across regions. Experiments on the IEEE 30-bus and IEEE 118-bus systems show that VFedA achieves similar performance to centralized detection methods and significantly outperforms HFL methods while also preserving data privacy. Ablation and sensitivity analyses confirm the effectiveness of the alignment module and reveal its optimal parameter settings. Overall, VFedA offers an effective, privacy-preserving, and scalable solution for FDIA detection in large-scale power systems.

Importantly, federated learning should not be interpreted as providing absolute privacy. In VFedA, raw regional measurements remain at the clients; only learned representations and the gradients associated with the relational alignment loss are exchanged. These representations are not mathematically guaranteed to be information-theoretically non-invertible. A sufficiently powerful adversary with auxiliary information could potentially attempt representation inversion or inference. While VFedA prevents direct exposure of raw measurement data under the considered honest-but-curious collaboration setting, this does not provide complete protection against representation inference attacks.

## Data Availability

The original contributions presented in this study are included in the article. Further inquiries can be directed to the corresponding author.

## References

[1] Ahsan F., Dana N., Sarker S., Li L., Muyeen S., Ali M. (2023). Data-driven next-generation smart grid towards sustainable energy evolution: Techniques and technology review. Prot. Control Mod. Power Syst..

[2] Zhang Z., Liu M., Sun M., Deng R., Cheng, Niyato D., Chow M., Chen J. (2024). Vulnerability of machine learning approaches applied in IoT-based smart grid: A review. IEEE Internet Things J..

[3] Liu M., Teng F., Zhang Z., Ge, Sun M., Deng R., Chen J. (2024). Enhancing cyber-resiliency of DER-based smart grid: A survey. IEEE Trans. Smart Grid.

[4] Huang X., Zhang Z., Yang Z., Ren C., Yau D., Deng R. (2026). FedCLD: A Federated Contrastive Learning Approach for Detecting Stealthy Attacks on Smart Grid with Unlabeled Data. IEEE Internet Things J..

[5] Zhang Z., Deng R., Yau D., Cheng, Chow M. (2022). Security enhancement of power system state estimation with an effective and low-cost moving target defense. IEEE Trans. Syst. Man Cybern. Syst..

[6] Liang G., Weller S., Zhao J., Luo F., Dong Z. (2017). The 2015 Ukraine Blackout: Implications for False Data Injection Attacks. IEEE Trans. Power Syst..

[7] Nand K., Zhang Z., Hu J. (2025). A Comprehensive Survey on the Usage of Machine Learning to Detect False Data Injection Attacks in Smart Grids. IEEE Open J. Comput. Soc..

[8] Kausar F., Deo S., Hussain S., Haque Z. (2024). Federated Deep Learning Model for False Data Injection Attack Detection in Cyber Physical Power Systems. Energies.

[9] Saber A., Farag H., Youssef A., Kundur D. (2025). A Model-Independent Trojan Attack on Deep Learning-Based FDIA Detection in Smart Grid Protection Systems. IEEE Trans. Instrum. Meas..

[10] Kesici M., Pal B., Yang G. (2024). Detection of False Data Injection Attacks in Distribution Networks: A Vertical Federated Learning Approach. IEEE Trans. Smart Grid.

[11] Wen J., Zhang Z., Lan Y., Cui Z., Cai J., Zhang W. (2022). A survey on federated learning: Challenges and applications. Int. J. Mach. Learn. Cybern..

[12] Kececi C., Davis K., Serpedin E. (2025). Federated Learning-Based Distributed Localization of False Data Injection Attacks on Smart Grids. IEEE Syst. J..

[13] Liu Y., Ning P., Reiter M.K. (2011). False data injection attacks against state estimation in electric power grids. ACM Trans. Inf. Syst. Secur..

[14] Irfan M., Sadighian A., Tanveer A., Al-Naimi S., Oligeri G. (2024). A survey on detection and localisation of false data injection attacks in smart grids. IET Cyber Phys. Syst. Theory Appl..

[15] Ahmad R., Alkhammash E. (2024). Online Adaptive Kalman Filtering for Real-Time Anomaly Detection in Wireless Sensor Networks. Sensors.

[16] Musleh A., Chen G., Dong Z. (2020). A Survey on the Detection Algorithms for False Data Injection Attacks in Smart Grids. IEEE Trans. Smart Grid.

[17] Sayghe A., Hu Y., Zografopoulos I., Liu X., Dutta R., Jin Y., Konstantinou C. (2020). Survey of machine learning methods for detecting false data injection attacks in power systems. IET Smart Grid.

[18] Wang Y., Lu Z., Ma J., Jin Q. (2025). Locational False Data Injection Attack Detection in Smart Grid Using Recursive Variational Graph Autoencoder. IEEE Internet Things J..

[19] Hasan M.S., Ismail M.I., Ibrahem M.I., Fouda M. Cyberattack Detection in Smart Grids: A Survey of Machine Learning Approaches and Challenges. Proceedings of the 2026 Intermountain Engineering, Technology and Computing (IETC).

[20] Wakwinga E. A Study on False Data Injection Attack Detection and Localization in Smart Grids Using ST-GNN with Adaptive Mechanisms. Proceedings of the 2025 IEEE 17th International Conference on Computational Intelligence and Communication Networks (CICN).

[21] Behnam R., Baghaee H., Gharehpetian G. (2026). GCN-LSTM-based detection of data integrity cyberattacks on STATCOM. Comput. Electr. Eng..

[22] Qin Y., Huang B., Tian J., Zhang Y., Wang L., Li Y. (2026). Distributed false data injection attack detection and localization based on heterogeneous data feature extraction and reconstruction of graph convolution network for microgrid. Sustain. Energy Technol. Assess..

[23] Li P., Dai Z., Wang M., Wang Z. (2025). Power system state estimation based on graph contrastive learning. Expert Syst. Appl..

[24] Kairouz P., McMahan H. (2021). Advances and Open Problems in Federated Learning. Found. Trends Mach. Learn..

[25] Shrestha R., Chamana M., Adeyanju O., Mohammadpourfard M., Bayne S. (2025). Detection and Localization of the FDI Attacks in the Presence of DoS Attacks in Smart Grid. Smart Cities.

[26] Lin C., Zhou Q., Wang Z., Fan X., Xu Y., Xu Y. (2025). Detection of False Data Injection Attacks: A Protected Federated Deep Learning Based on Encryption Mechanism. Comput. Mater. Contin..

[27] Choubey M., Chaurasiya R., Yadav J. (2025). Contrastive learning for efficient anomaly detection in electricity load data. Sustain. Energy Grids Netw..

[28] Ruan J., Fan G., Zhu Y., Liang G., Zhao J., Wen F. (2023). Super-Resolution Perception Assisted Spatiotemporal Graph Deep Learning Against False Data Injection Attacks in Smart Grid. IEEE Trans. Smart Grid.

[29] Huang X., Zhang Z. (2026). Self-Supervised Spatio-Temporal Contrastive Learning for FDIA Detection in the Smart Grid. IEEE/CAA J. Autom. Sin..

